# Lisinopril-Induced Angioedema in a Patient with Plasma Prekallikrein Deficiency

**DOI:** 10.1055/s-0040-1701238

**Published:** 2020-01-23

**Authors:** Swapan K. Dasgupta, Stefanie Rivera, Perumal Thiagarajan

**Affiliations:** 1 Department of Pathology, Baylor College of Medicine, Houston, Texas, United States; 2 Department of Medicine, Baylor College of Medicine, Houston, Texas, United States; 3 Center for Translational Research on Inflammatory Diseases (CTRID), Michael E. DeBakey Veterans Affairs Medical Center, Houston, Texas, United States

**Keywords:** bradykinin, prekallikrein, kininogen

## Abstract

Angiotensin-converting enzyme (ACE) inhibitors are extensively prescribed to treat patients with hypertension, congestive heart failure, and diabetic nephropathy. A small fraction of these patients (approximately 0.7%) develop angioedema, manifested by swelling of the lips and oropharynx. Angioedema of oropharynx is a medical emergency that can lead to asphyxiation and death. The angioedema is due to bradykinin generated from high molecular weight kininogen by kallikrein, which is derived from plasma prekallikrein by action of the factor XIIa, factor Xia, or prolylcarboxypeptidase. Bradykinin induces vasodilation and increased vascular permeability. ACE is the major degrading enzyme of bradykinin in the intravascular department. ACE inhibitors inhibit the proteolytic inactivation of bradykinin. We report a patient with oropharyngeal angioedema associated with an ACE inhibitor with complete absence of plasma prekallikrein due to homozygous mutation (Ser97PhefsTer173).

A 67-year-old veteran came to our emergency department because of the sudden onset of tongue swelling and the inability to swallow saliva. His past medical history included type 2 diabetes mellitus, essential hypertension, and plasma prekallikrein deficiency. He had no itching or previous history of allergic reaction. He had been previously diagnosed to have plasma prekallikrein deficiency as his PTT had ranged from 90 to <120 seconds on different occasions (normal <35 seconds) The plasma kallikrein level was less 3% by coagulation assays. No immunoreactive prekallikrein was detected in the plasma by western blot ( Fig. 1 ). All other coagulation factors had been in the normal range, including factor XII. He had never had a bleeding diathesis. He started taking lisinopril for hypertension 3 months before the onset of angioedema. His physical examination was unremarkable except for oropharyngeal angioedema. He received intravenous dexamethasone, ranitidine, and diphenhydramine, and his symptoms resolved over several hours.

**Fig. 1.**
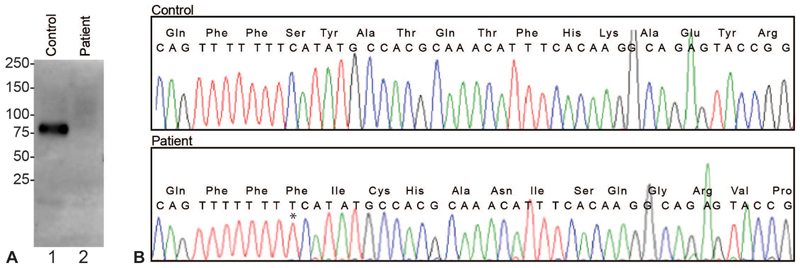
Mutation in prekallikrein gene. ( **A** ) Immunoblot of plasma. Two microliter of control and patient was electrophoresed and blotted with an antibody to plasma prekallikrein (clone 13G11 from Invitrogen). ( **B** ) Sequencing traces of PCR amplified exon 5 depicting the homozygous single nucleotide (*) insertion in codon 132, leading to frameshift all of subsequent codons. PCR, polymerase chain reaction.

Deoxyribonucleic acid (DNA) was extracted from peripheral blood leukocytes and all 15 exons of prekallikrein gene *(KLKB1)* were amplified by polymerase chain reaction (PCR) using genomic DNA as the template and sequenced. He had a homozygous single nucleotide insertion of thymine in codon 132, in exon 5, that caused amino acid change (Ser to Phe) and frameshift of subsequent codons resulting in a premature stop at codon 173 ( Fig. 1 ). To our knowledge, this mutation has never been described before. This exon codes for the apple 2 domain and a previously described amino acid substitutions (Asn to Ser in codon 124) were also present. 1

Angiotensin-converting enzyme (ACE) not only catalyzes the conversion of angiotensin I to II but also degrades plasma bradykinin (which induces vasodilation and increased vascular permeability) by proteolysis to inactive metabolites 2 3 . Substantial increase in bradykinin levels during acute attacks has been shown in patients with ACE-induced angioedema. 4 Occurrence of angioedema in a patient with complete deficiency of plasma prekallikrein shows other possible enzymes capable of liberating bradykinin such as factor XIIa, plasmin, or tissue kallikreins may also play a role in generating bradykinin during ACE inhibitor therapy ( Fig. 2 ). Mutation of the angiopoietin-1 gene ( *ANGPT1* ) can also induce angioedema independent of bradykinin. 5 These enzyme or proteins can be possible etiologies for angioedema independent of plasma kallikrein. In humans, plasma prekallikrein is coded by a single gene *KLKB1* , while tissue kallikreins are a family of 15 closely related serine proteases ( *KLK1-15* ). At least two tissue kallikreins, *KLK1* and *KLK2* , can generate bradykinin from low molecular weight kininogen. 6 Salivary secretion is a rich source of tissue kallikreins. The oropharynx with abundant saliva is the most common site of angioedema associated with ACE. It is pertinent to note that bradykinin, generated in the plasma during plasmapheresis or during infections, is associated with hypotension but not angioedema. 7 8 These clinical observations raise the possibility that bradykinin generated by tissue kallikrein present in the salivary secretions may have a causal role in oropharyngeal angioedema in patients receiving ACE inhibitors.

**Fig. 2.**
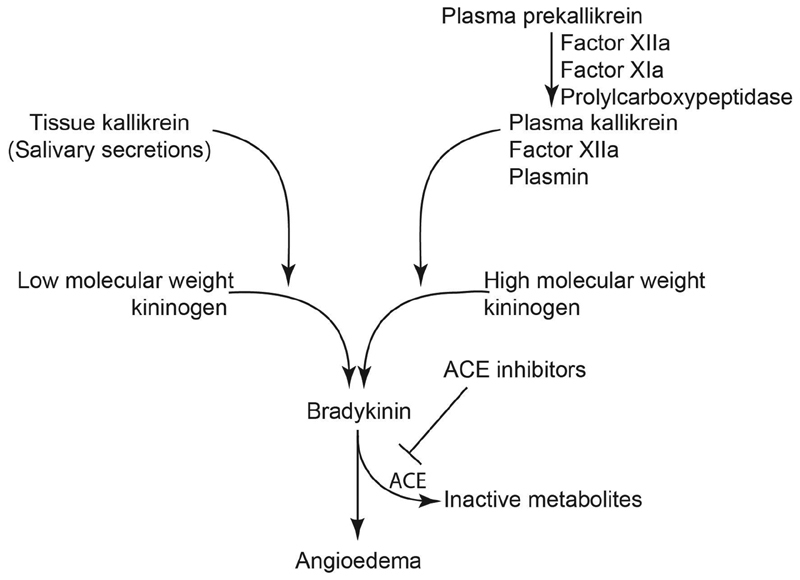
The kallikrein proteolytic cascade. The preferred substrate for plasma kallikrein is high molecular weight kininogen while tissue kallikreins metabolize low and high molecular weight kininogen.
